# Household and climate factors influence *Aedes aegypti* presence in the arid city of Huaquillas, Ecuador

**DOI:** 10.1371/journal.pntd.0009931

**Published:** 2021-11-16

**Authors:** James L. Martin, Catherine A. Lippi, Anna M. Stewart-Ibarra, Efraín Beltrán Ayala, Erin A. Mordecai, Rachel Sippy, Froilán Heras Heras, Jason K. Blackburn, Sadie J. Ryan

**Affiliations:** 1 Quantitative Disease Ecology and Conservation (QDEC) Lab, Department of Geography, University of Florida, Gainesville, Florida, United States of America; 2 Emerging Pathogens Institute, University of Florida, Gainesville, Florida, United States of America; 3 Institute for Global Health & Translational Science, SUNY Upstate Medical University; 4 Department of Medicine, SUNY Upstate Medical University, Syracuse, New York, United States of America; 5 InterAmerican Institute for Global Change Research (IAI), Montevideo, Uruguay; 6 Universidad Técnica de Machala, Machala, Ecuador; 7 Biology Department, Stanford University, Stanford, California, United States of America; 8 Spatial Epidemiology and Ecology Research Laboratory, Department of Geography, University of Florida, Gainesville, Florida, United States of America; Centers for Disease Control and Prevention, Puerto Rico, UNITED STATES

## Abstract

Arboviruses transmitted by *Aedes aegypti* (e.g., dengue, chikungunya, Zika) are of major public health concern on the arid coastal border of Ecuador and Peru. This high transit border is a critical disease surveillance site due to human movement-associated risk of transmission. Local level studies are thus integral to capturing the dynamics and distribution of vector populations and social-ecological drivers of risk, to inform targeted public health interventions. Our study examines factors associated with household-level *Ae*. *aegypti* presence in Huaquillas, Ecuador, while accounting for spatial and temporal effects. From January to May of 2017, adult mosquitoes were collected from a cohort of households (n = 63) in clusters (n = 10), across the city of Huaquillas, using aspirator backpacks. Household surveys describing housing conditions, demographics, economics, travel, disease prevention, and city services were conducted by local enumerators. This study was conducted during the normal arbovirus transmission season (January—May), but during an exceptionally dry year. Household level *Ae*. *aegypti* presence peaked in February, and counts were highest in weeks with high temperatures and a week after increased rainfall. Univariate analyses with proportional odds logistic regression were used to explore household social-ecological variables and female *Ae*. *aegypti* presence. We found that homes were more likely to have *Ae*. *aegypti* when households had interruptions in piped water service. *Ae*. *aegypti* presence was less likely in households with septic systems. Based on our findings, infrastructure access and seasonal climate are important considerations for vector control in this city, and even in dry years, the arid environment of Huaquillas supports *Ae*. *aegypti* breeding habitat.

## Author summary

Mosquito transmitted infectious diseases are a growing concern around the world. The yellow fever mosquito (*Aedes aegypti*) has been responsible for recent major outbreaks of disease, including dengue fever and Zika. This mosquito prefers to bite humans and lay its eggs in artificial containers such as water tanks and planters. This makes *Ae*. *aegypti* well suited to become established in growing urban areas. Controlling these mosquitoes has been an important way to reduce the risk of disease transmission. Studies that are undertaken to understand local factors that contribute to the continued survival of the mosquito can be used to inform control practices. We conducted a study in the largest Ecuadorian city on the border of Peru where we collected adult mosquitoes from houses and surveyed household members about their behaviors, perceptions, and housing infrastructure associated with the mosquito vector. Mosquitoes were most numerous in weeks with high temperatures and a week after increased rainfall. We found that houses that had unreliable water service were more likely have mosquitoes present, while houses that used septic systems were less likely to have mosquitoes present.

## Introduction

Arboviral diseases are an increasing global concern [1], exemplified by the large health burden of dengue fever, where 58.4 million cases are reported annually worldwide [2]. The yellow fever mosquito (*Aedes aegypti*) is the primary vector of dengue virus (DENV) and other medically important arboviruses such as chikungunya, yellow fever, and Zika [1,3]. This mosquito species is well-adapted to urban environments, as it is an anthropophilic container breeder that readily exploits the built environment for ovipositional sites [4]. Globally, increasing trends in urbanization, international trade, and travel have further facilitated the spread and establishment of *Ae*. *aegypti* over the years, and consequently, the diseases it transmits [5].

Vector control remains a primary strategy in controlling arboviral diseases [6], largely due to limited options for clinical treatment, and with the exception of yellow fever, lack of widely marketable vaccines [7,8]. Although an integral tool in mosquito-borne disease management, there is often a need to target mosquito control efforts, both to improve their effectiveness and to conserve limited public health agency resources. *Aedes aegypti* mosquitoes are sensitive to abiotic environmental factors such as climate, including temperature, rainfall and relative humidity, all of which can be highly variable at fine spatial scales and influenced by features of the built environment [9]. Further, the anthropophilic nature of *Ae*. *aegypti* mosquitoes makes them sensitive to certain human behaviors and activities, [10] such as household water storage practices or use of insecticides. Social-ecological factors that modulate vector populations represent potential targets for mosquito control interventions. However, it has become increasingly apparent that local drivers of vector populations can vary greatly in space and time [11,12], which has significant implications for the planning of successful public health vector control programs. It is therefore important to examine both the social and ecological components of a local environment to understand sub-city risks of vector presence, and potential leverage points for intervention. Thus, local studies conducted at appropriate spatiotemporal scales are necessary to assess place-specific drivers and inform interventions [13].

The South American country of Ecuador has a history of high arboviral disease burden [14,15]. Yellow fever dominated during early 20th century, and at the beginning of the 21st century dengue fever emerged as the principal mosquito-borne disease, coinciding with a decline in malaria cases [16,17]. More recently, the introduction of new viruses, such as chikungunya and Zika, has resulted in major epidemics throughout the region [18]. There have been multiple studies in recent years that have examined the influence of climate and social-ecological factors on *Ae*. *aegypti* in coastal Ecuador [10,14,19–24]. These studies typically feature analyses conducted with data aggregated to neighborhoods or census blocks, and much of the literature is primarily focused on the city of Machala, Ecuador, located in El Oro province on the country’s southern coast. Machala is an urban center with a steppe climate (BSh) per the Köppen climate classification system, indicating conditions intermediate between desert and humid climates. Studies conducted in Machala have repeatedly found that both climate and social-ecological factors influence the presence of both immature and adult *Ae*. *aegypti* in neighborhoods, providing local health authorities with valuable information for guiding local mosquito control campaigns [10,19–24].

To date, there have been no studies examining the impacts of climate and social-ecological system (SES) on *Ae*. *aegypti* in the desert climates of Ecuador. Huaquillas is located in El Oro province, on Ecuador’s southern border with Peru. Huaquillas has a distinct arid climate compared to other coastal locations in Ecuador where previous vector-borne disease studies have typically been conducted. Further, the position of Huaquillas near an international border presents an additional opportunity for studying disease vectors in a novel SES within Ecuador. Differences in environment, socio-economic status, access to healthcare, healthcare practices, and vector control practices between countries can impact the burden of disease, abundance of vectors, and infection levels of vectors, potentially leading to drastic differences in the SES of Huaquillas, relative to other communities in Ecuador [25,26]. Cities along borders, such as Huaquillas, may also have larger migrant populations than other cities, or may be a through-way for migrating populations, who can serve as reservoirs of vector-borne disease [27,28]. Thus, describing the characteristics that influence vector presence within Huaquillas may help in developing intervention strategies that are tailored to meet the challenges of delivering effective mosquito control in a unique SES.

This study aims to describe the climatic and social-ecological aspects of household-level *Ae*. *aegypti* presence in the arid border city of Huaquillas. These insights can lead to a better understanding of how this vector-borne disease system functions and where potential levers of control might be found. The knowledge gained can help inform intervention decisions in Huaquillas and other similar settings. Additionally, this study, in combination with similar local-scale studies in the region, can help answer questions relating to scale and heterogeneity in arboviral disease systems.

## Methods

### Ethical review

This study protocol was reviewed and approved by Institutional Review Boards (IRBs) at SUNY Upstate University, the Luis Vernaza hospital in Guayaquil, Ecuador, and the Ministry of Health of Ecuador. Prior to the start of the study, adult participants (≥ 18 years of age) engaged in a written informed consent (conducted in Spanish). To protect the privacy of participants, data were de-identified prior to use in any of the analyses conducted in this study.

### Study site

**Fig 1:**
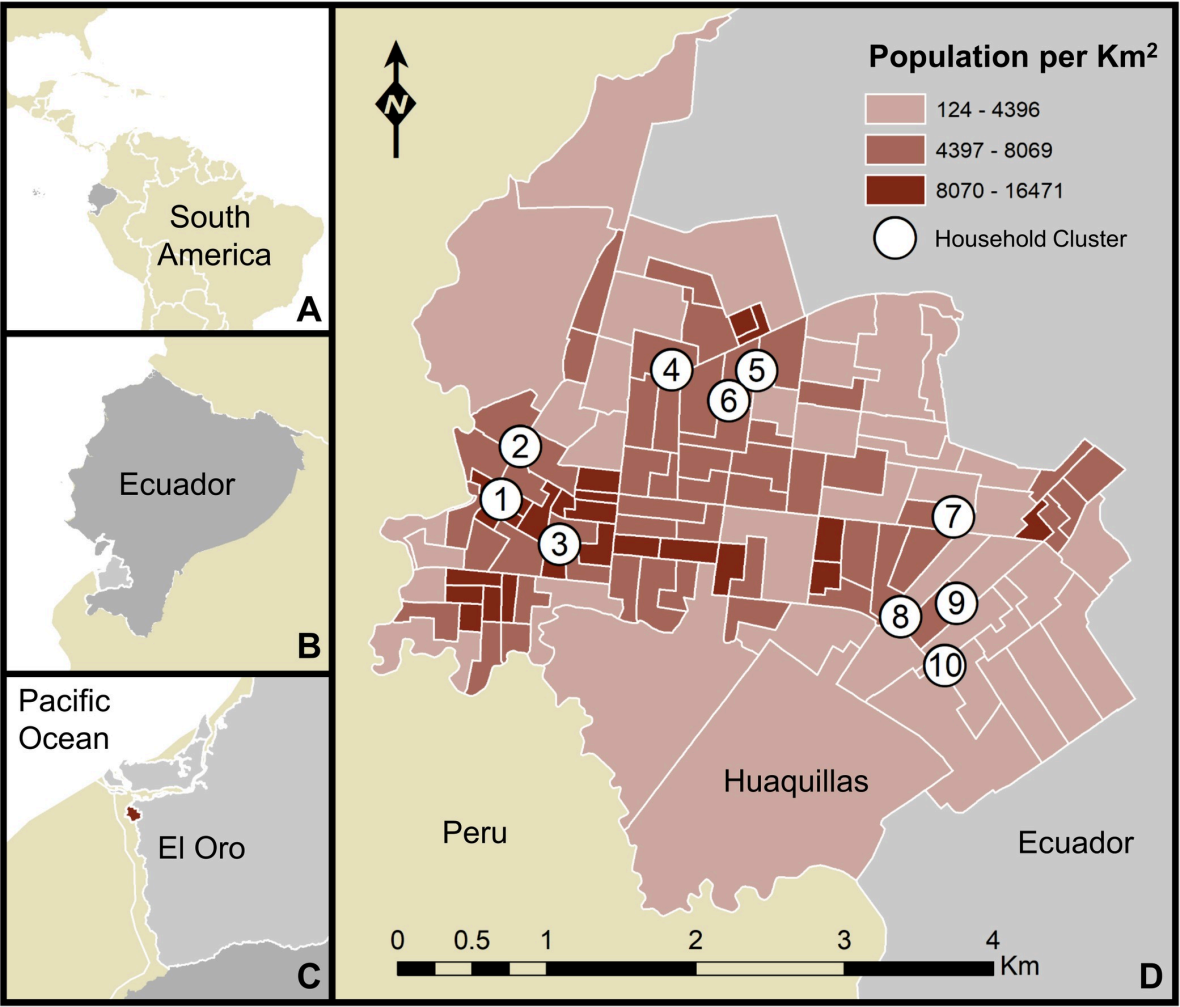
Map of the study area. Huaquillas is located within South America, Ecuador, and El Oro province. The map includes household cluster locations where sampling took place (white circles denote areas where up to 5 houses were sampled, but precise household locations are not shown, to protect identities) and the population density of Huaquillas at the census tract level. A) location of Ecuador, B) location of El Oro province, C) location of Huaquillas (red), D) Huaquillas population and sampling sites. This figure was produced in ArcMap 10.6.1 (ESRI, Redlands, CA) using shapefiles freely available from the Natural Earth dataset ver. 4.1.0 (naturalearthdata.com) and georeferenced census data (2010) provided by the Ecuadorian National Institute of Statistics and Census (INEC) and edited by JLM.

Huaquillas is a coastal city located in southern Ecuador’s El Oro province, with a population of 48,285 (Fig 1) [29]. Situated in a low-lying coastal region (3°28’33”S, 80°13’33”W; 15m elevation), the city is highly suitable for *Ae*. *aegypti*, as underscored by endemic transmission of DENV, which has led to major outbreaks of dengue fever in recent years [22]. Regionally, dengue outbreaks in coastal Ecuador tend to peak during the hot and rainy season, which typically begins in January [30]. Huaquillas is the primary crossing at Ecuador’s southern border with Peru, and became a major hub of transit and economic exchange between the two countries, following an agreement to open the border in the 1990’s [31,32]. In terms of total migration, Huaquillas is the third most active city in Ecuador, with 622,405 arrivals and departures annually [31]. Increased binational cooperation with Peru and the relaxation of trade and travel barriers in recent decades has contributed to increased urban development at the border [33].

**Fig 2:**
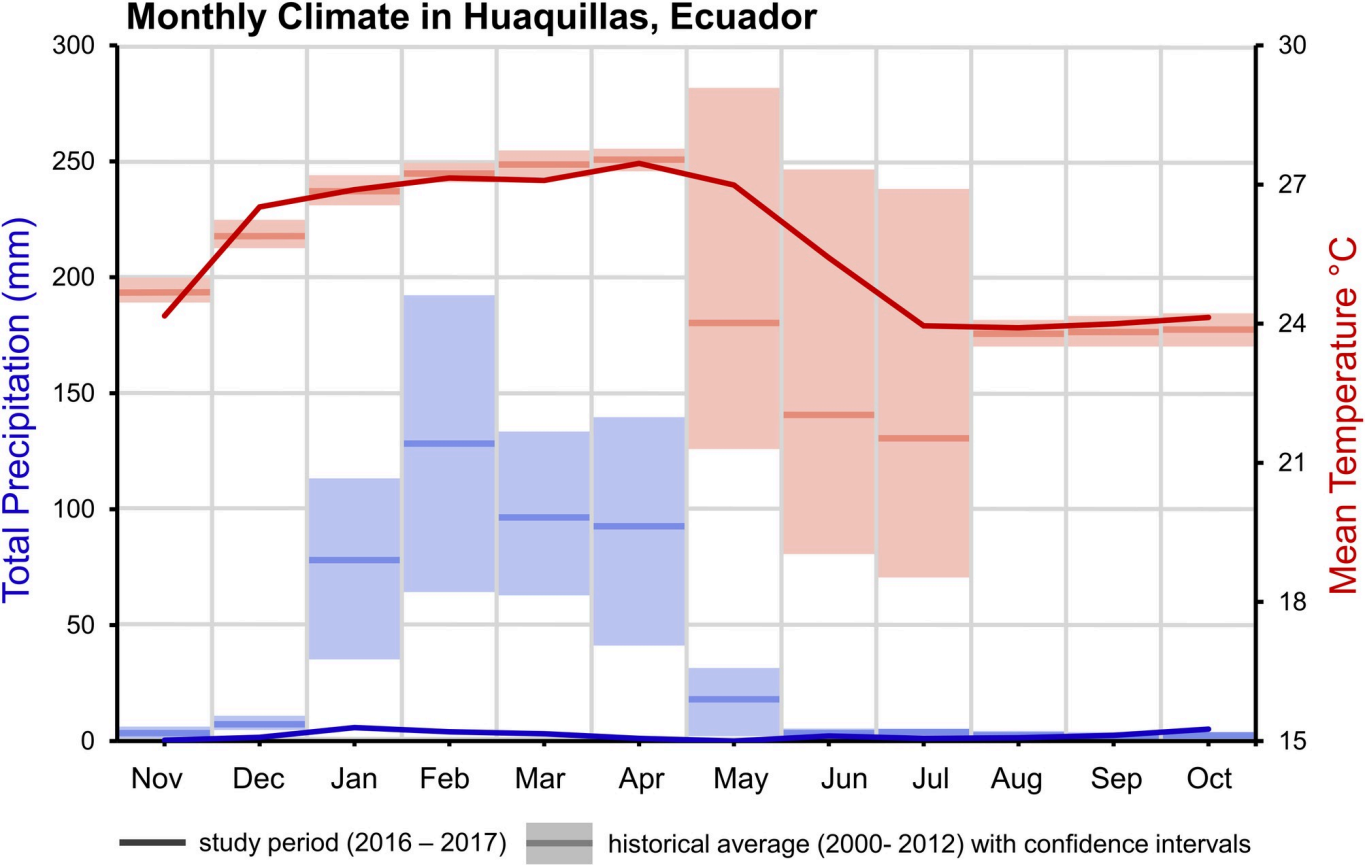
Climate in Huaquillas, Ecuador. Monthly mean temperature in red and total monthly precipitation as blue. Solid lines represent the climate during the study period (2016–2017) while box plots represent the climatology from 2000–2012 (historical monthly averages and 95% confidence intervals).

Huaquillas has a hot desert climate (BWh) per the the Köppen climate classification system [34]. While desert climates are often characterized by limited precipitation, intense sunlight, and little vegetation, actual conditions can vary greatly by place. Huaquillas experiences a monsoon season which occurs during the first half of the year, with monthly total rainfall peaking in February at 128 mm (Fig 2). Increased precipitation coincides with high temperatures. Annual mean temperature ranges from 21.6–27.6°C. Minimum daily temperatures range from 18.8–22.3°C annually and maximum daily temperatures range from 25.8–33.0°C annually. Fig 2 shows a comparison of historic long-term average climate data (2000–2012) provided by the National Institute of Meteorology and Hydrology (INAMHI), Ecuador, and the data provided by INAMHI for the study period (2016–2017).

### Data sources

All data cleaning and processing was conducted in R version 3.5.1 [35].

#### Climate data

The National Institute of Meteorology and Hydrology (INAMHI), Ecuador, provided hourly climate data (March 2016—December 2017) from an automatic weather station in Huaquillas. These data included minimum, maximum, and mean temperature, and total daily precipitation. Hourly climate data were aggregated to weekly measures to match the resolution of entomological sampling events for statistical analyses. Hourly precipitation readings during that period were summed for weekly precipitation, and hourly temperature data were used to derive daily mean, minimum, and maximum temperatures.

#### Participating households

Households were invited to participate in this study using a semi-random selection process to capture clusters of households facing similar risk of exposure to *Ae*. *aegypti* mosquitoes. Ten central households, representing the center of clusters, were chosen throughout Huaquillas to maximize geographic coverage. Each cluster consisted of the central household, and up to five additional households randomly enrolled into the study from within a 250m radius of the central household, a distance that approximates the flight range of *Ae*. *aegypti* [36]. Enrolled houses were georeferenced onsite using handheld global positioning system (GPS) units and given specific house and cluster codes. The resulting cohort of households was a favorable balance between sufficiently accounting for the heterogeneity of the urban environment in Huaquillas and the logistical feasibility of regularly visiting the households for data collection.

#### Household mosquito samples

Teams of field technicians visited households biweekly during January—May 2017 to collect adult mosquitoes from households using Prokopak backpack aspirators. During a sampling event, all participating households in a cluster were surveyed for adult mosquitoes. Not all clusters were sampled in a given sampling day for logistical reasons, and consequently individual households were sampled for mosquitoes at 2–3 week intervals. Mosquito collections were conducted during the daytime. The entomological sampling protocol consisted of one technician operating the backpack aspirator and sampling the intradomicile (i.e. within the home) for 20 minutes and the peridomicile (i.e. courtyard or patio associated with home) for 10 minutes. Each room within a given household was sampled, starting at the floor, sampling under furniture, and working up to the ceiling. Adult mosquitoes collected via aspiration were stored on ice in a cooler and were transported to the entomology lab at the Universidad Técnica de Machala, Ecuador, 74km by vehicle, where specimens were enumerated, sorted by sex, and identified (i.e., *Ae*. *aegypti* and other). Due to low counts (e.g. <10), household *Ae*. *aegypti* abundance was summarized as binary outcomes. Sampling events were classified as “positive” if female *Ae*. *aegypti* mosquitoes were present in a given household, and the proportion of positive households for each week was calculated for statistical analysis to account for temporally discontinuous abundance sampling [37].

#### Household surveys

Upon study enrollment, field technicians administered a survey questionnaire to the head of household (HOH); the complete survey tool is available in both English and Spanish elsewhere [38]. Using the survey tool, technicians collected data on household demographics, HOH occupation, household expenditures, access to public services, knowledge and perceptions of mosquito-borne disease, and mosquito control and prevention practices [38]. Field personnel concurrently performed an on-site visual assessment of housing structures and conditions, following protocols used in previous studies conducted in the region [10,38]. Collectively, data recorded during household surveys comprise the social-ecological system (SES) variables used in statistical analyses.

### Statistical analyses

#### Household mosquito samples

To assess climate lags on household-level presence of female *Ae*. *aegypti* in the study group, at a monthly scale, only households with sampling events in all months were included. For each household, if multiple collections occurred in a month, one collection record was randomly selected to represent *Ae*. *aegypti* presence in that month.

Cross correlation function (CCF) plots were visualized in R version 3.5.1 [35] for *Ae*. *aegypti* presence and climate variables (precipitation, mean, maximum, and minimum temperature) lagged from 0 to 6 weeks. Shapiro-Wilk normality tests indicated that the mosquito presence data did not violate assumptions, while the temperature and precipitation variables did [39]. We therefore ran Spearman’s rank correlation tests on the lagged climate variables [40] (S1 Fig). Statistically significant climate indicators were used to construct a beta regression model (link = logit, link.phi = identity) in R with the package “betareg” [41,42] to capture the relationship between climate and the proportion of positive households in sampling clusters [41,43]. Model residuals were checked for normality with the Shapiro-Wilk test, and for autocorrelation with the autocorrelation function in R.

#### Social-ecological system (SES) models of household factors and Aedes aegypti presence

Associations between survey responses and household female *Ae*. *aegypti* presence during the study period were measured using univariate statistical tests. Questions that addressed social-ecological factors hypothesized to be important for vector population dynamics at this study site (based on previous studies in this region) were selected from the full household survey for analysis [10,20,38,44]. Hypothesized factors included water storage practices, building materials, and economic status. Data inclusion criteria for SES models were stringent, and survey questions that had a low rate of response were excluded to minimize observations discarded due to missing data. Questions that had the same response for nearly every observation were also excluded because they offer little ability to differentiate between houses with and without *Ae*. *aegypti*. Households were required to have mosquito samples in all months of the study; those with more than one sampling event per month had one event randomly selected for inclusion. Intraclass correlation (ICC) values for household data were near zero (ICC = 0.01), indicating that the clustered study design did not greatly impact variance in the data, thus precluding the need for a mixed-effects modeling structure [45,46]. Univariate proportional odds logistic regression was used to assess differences in survey responses by monthly household *Ae*. *aegypti* presence [47–49]. Models to assess the relationship between ordinal mosquito sampling (i.e. the number of sampling events where *Ae*. *aegypti* were detected) and household variables were built in R with the “MASS” package [50], using the default options of the ‘polr’ function to perform proportional odds logistic regressions [49,51]. The Brant test [52] was used to confirm that proportional odds models did not violate the parallel regression assumption [51], and was implemented with the “brant” package in R [53].

## Results

**Fig 3:**
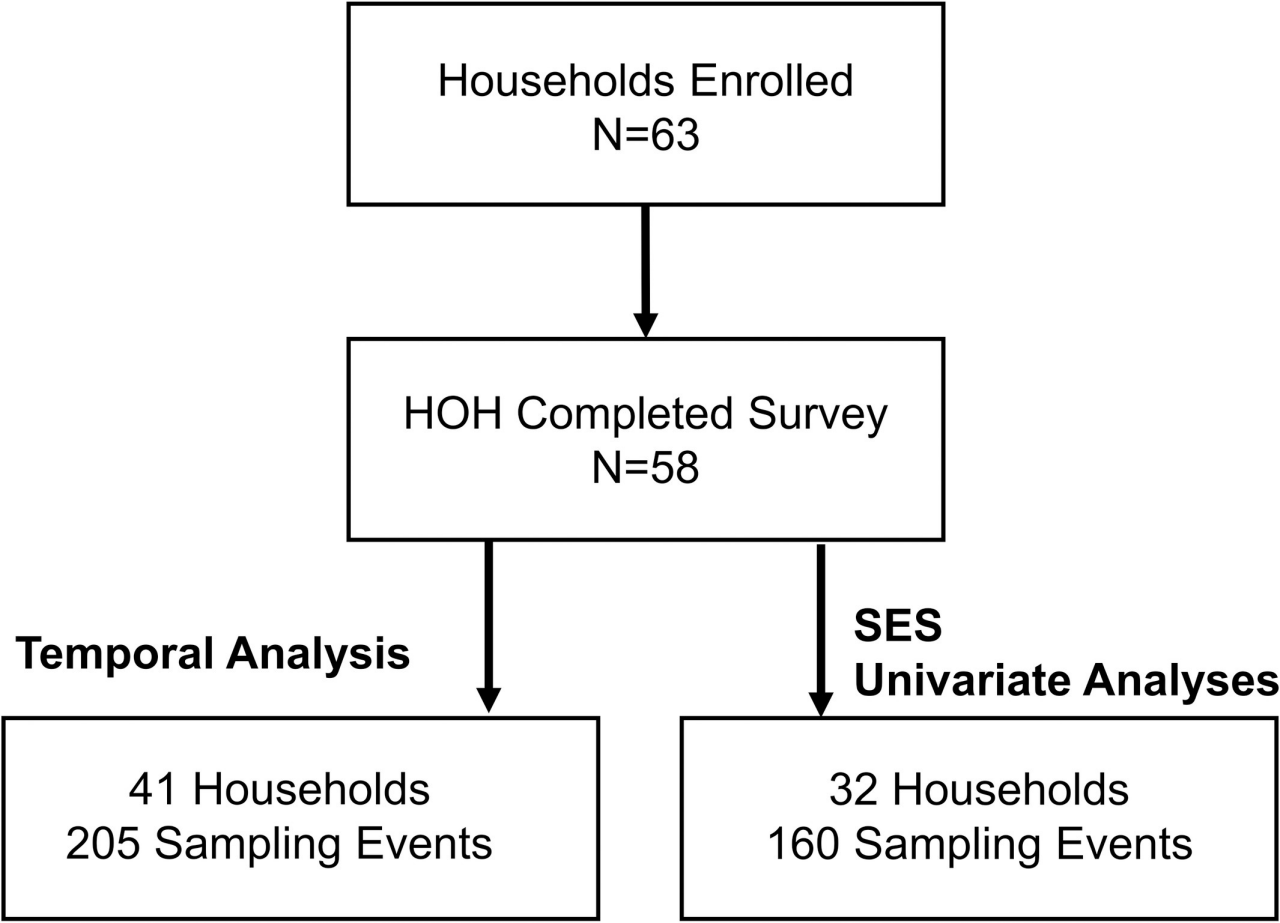
Diagram of household enrollment and data collection for the cluster study in Huaquillas, Ecuador. More restrictive criteria for data inclusion were used for statistical analysis of SES factors.

Sixty-three households participated in the study, and of these, fifty-eight heads of household (HOHs) (92%) responded to the survey (Fig 3). The average distance between households within clusters was 86 m. A total of 458 mosquito collections occurred over 10 individual weeks of sampling. For assessments of climate impacts and temporal signals, 41 houses (65%) with 205 mosquito sampling events met the criteria for inclusion. For assessments of household characteristics and SES models, 32 houses (51%) with 160 mosquito sampling events met the criteria for inclusion (S1 Table).

### Climate

Weekly *Ae*. *aegypti* female presence was significantly correlated with precipitation, at a 1-week lag (rho = 0.84, p = 0.002), 3 weeks (rho = 0.73, p = 0.018), and 5 weeks (rho = 0.69, p = 0.026). Weekly *Ae*. *aegypti* female presence was also significantly correlated with current week mean temperature (rho = 0.624, p = 0.012), and negatively correlated with maximum temperature on a 5-week lag (rho = 0.70, p = 0.03). Minimum temperatures during this period were not significantly correlated with *Ae*. *aegypti* presence at any lag.

A beta regression of the proportion of households with female *Ae*. *aegypti* presence as a function of the significant climate lags had a pseudo R^2^ of 0.77 (p = 0.023), and precipitation at a week lag (β = 1.91, p = 0.03) and mean temperature of the week (β = 0.69, p = 0.008) remained individually significant and positive in the model.

### Temporal patterns of Ae. aegypti presence

**Fig 4:**
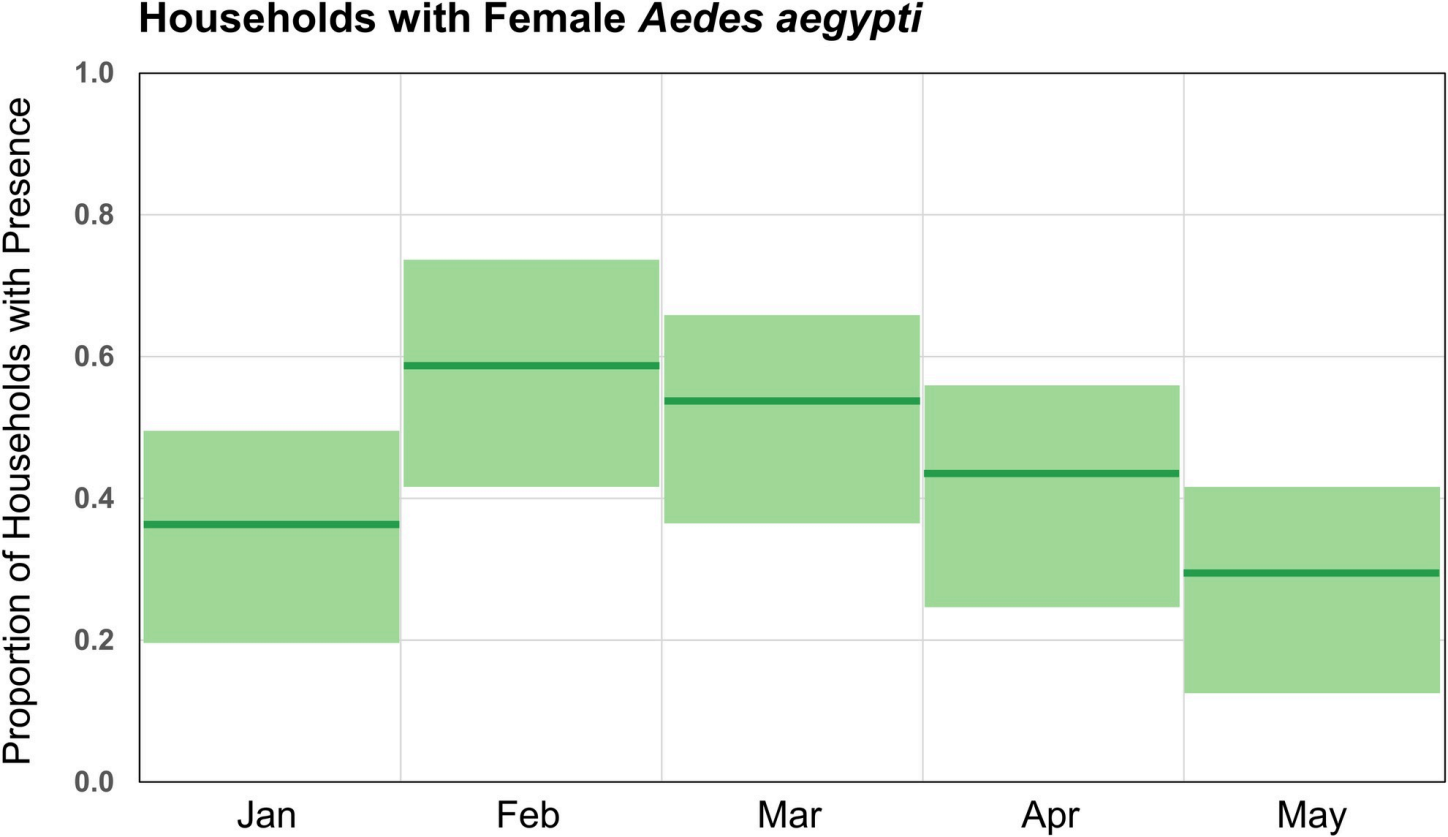
Female *Aedes aegypti* presence in households in Huaquillas, Ecuador, by month in 2017. Month proportions and 95% confidence intervals for 41 households used in this dataset. Chi-squared test for difference in proportions: 9.56, p = 0.0484).

The proportion of positive households was highest in February and lowest in May (Fig 4). In January, the proportion of positive households was 0.38; this increased to 0.59 in February before steadily declining. The monthly differences were significant (χ^2^ = 9.564, p = 0.048), but a post hoc Fisher’s exact test did not identify specific month-to-month differences (S2 Table).

### Univariate tests for SES factors

All respondents considered dengue fever to be a severe disease and correctly answered questions about the transmission cycle of the disease, so these variables were not included in this analysis. Univariate model parameter estimates are given in Table 1, in which the odds ratio (OR) denotes risk of exposure for OR>1, and OR<1 is protective against *Ae*. *aegypti* presence. Demographic variables and factors describing the physical conditions of households were not found to be significant predictors of female *Ae*. *aegypti* presence (Table 1). However, one of the four variables describing infrastructure and public services was significant; interruptions in piped water supply (OR = 4.78) was positively associated with mosquito presence. An additional infrastructure variable was on the threshold of statistical significance (p = 0.051), use of a septic tank (OR = 0.13), which had a negative association. Mosquito prevention methods, employment, and travel habits were not found to be significantly associated with household presence of *Ae*. *aegypti*.

**Table 1:** Household level social-ecological factors associated with female *Aedes aegypti* presence (AA) in homes in Huaquillas, Ecuador. Significant associations (p<0.05) are in bold.

SES Factor	HH^a^ (n = 32)	Estimate	St. Error	OR	p-value	95% CI
People Living in the House (mean)	4.5	0.15	0.16	1.16	0.348	0.85–1.59
Years Living in Neighborhood (mean)	7.75	0.09	0.07	1.10	0.159	0.97–1.25
Age of HOH (mean)	47.13	0.01	0.02	1.01	0.715	0.97–1.05
Female HOH	6	0.49	0.78	1.64	0.529	0.35–7.92
HOH Beyond Primary Education	15	0.41	0.64	1.50	0.525	0.43–5.39
HOH Makes Basic Income	7	0.92	0.73	2.50	0.209	0.60–10.89
Number of Rooms (mean)	2.69	0.33	0.37	1.39	0.363	0.68–2.90
Good Overall Condition	16	0.15	0.63	1.16	0.814	0.34–4.00
Good Flooring	14	0.74	0.65	2.10	0.251	0.60–7.67
Screens on Windows	7	-0.24	0.77	0.79	0.755	0.167–3.60
Shaded Patio	27	0.21	0.78	1.23	0.789	0.26–5.85
Abandoned Houses Nearby	22	-0.59	0.67	0.56	0.383	0.14–2.06
Unpaved Road	20	-0.16	0.63	0.85	0.797	0.24–2.96
Stores Water	24	0.69	0.75	1.99	0.356	0.46–8.91
**Water Interruptions**	8	1.56	0.78	4.78	0.044	1.09–24.13
**Uses Septic Tank**	3	-2.07	1.06	0.13	0.051	0.01–0.98
Biweekly Trash Collection	20	1.27	0.68	3.57	0.061	0.97–14.13
Outdoor Labor	13	-0.32	0.65	0.73	0.625	0.20–2.62
Indoor Service	16	0.04	0.63	1.04	0.953	0.30–3.57
Treated Water in Past 30 days	11	0.94	0.68	2.55	0.171	0.68–10.14
Uses Abate Larvicide	29	-1.08	0.98	0.34	0.270	0.04–2.36
Drains Standing water	30	-0.07	1.09	0.93	0.946	0.10–8.95
Closes Windows and Doors	30	0.82	1.11	2.28	0.457	0.23–22.79
HOH Works Outside of the City	7	0.35	0.74	1.43	0.630	0.33–6.19
Does Not Leave Neighborhood for Work	30	0.37	1.15	1.45	0.745	0.14–15.73

^a^ = Number of households with factor

## Discussion

In this study we investigated the drivers of household-level female *Ae*. *aegypti* presence in Huaquillas, Ecuador, to identify social-ecological conditions that promote potential arboviral disease risk to inform vector control and intervention strategies. Precipitation during the study period was anomalously low compared to long-term averages (Fig 1). In several recent studies, the role of drought in altering the way water storage occurs in urban landscapes has been highlighted as a potential key factor in *Ae*. *aegypti* habitat in urban environments [54–60]. Given this was a particularly dry year, in an already arid environment, the role of precipitation in the timing of *Ae*. *aegypti* presence may be different than in an average year. We found that the prior week’s precipitation was an important predictor of *Ae*. *aegypti* presence, in combination with the current week’s temperature. Whether the role of precipitation is emphasized or diminished in a dry year is likely mediated by human-driven water storage and use on the landscape. In outdoor, rain-filled habitats, accumulated precipitation can generate oviposition sites for *Ae*. *aegypti*, but extreme precipitation events can flush out those same larval habitats [61,62]. Thus, the relationship between precipitation and vector population size is not linear, and may depend more on the intensity of precipitation events [13,63]. In our study, *Ae*. *aegypti* presence was significantly correlated with precipitation lagged by 1, 3, and 5 weeks, individually, but when included in a model with temperature, the longer lags were no longer individually significant. In rain-filled habitats, precipitation events increase the suitability of larval habitats, prompting eggs of *Ae*. *aegypti* to hatch and begin development [64]. The 1-week lag in precipitation likely indicates sufficient humidity and moisture for mosquito activity in the current week, and perhaps serves to trigger egg hatching, but is likely too short a time-frame for development to flying adults. The 3-week and 5-week lags identified in this study are longer than the typical development time for *Ae*. *aegypti* [65], however in an arid environment such as this, larval habitat may dry after precipitation events, increasing the time necessary to develop [66,67]. In the urban environment, the timing and degree to which precipitation influences vectors is also highly modulated by the social-ecological environment [10,68]. The role of precipitation may be more identifiable when containers and buckets are visibly on household premises, but is diminished when alternative oviposition sites such as water storage tanks, cisterns, and other water infrastructure sites are available [54,69].

During the study period, mean temperatures in Huaquillas were within historical ranges (Fig 1). Laboratory studies of *Ae*. *aegypti* have found nonlinear relationships between mean temperature and physiological traits [70,71]. The weekly mean temperatures assessed in our climate analysis ranged from approximately 26 and 28°C. Biting, development, fecundity, and mortality have been shown to be positively correlated with mean temperatures within this range [72]. We found that weekly *Ae*. *aegypti* presence was significantly associated with mean temperatures of the same week. This captures the immediate effect of temperature on *Ae*. *aegypti* presence in households where people reside [73]. Outdoor temperatures higher than 21°C may drive *Ae*. *aegypti* indoors to reduce mortality [74]; indoor resting behavior is characteristic of *Ae*. *aegypti*, especially while processing blood meals [75], so sheltering in shade indoors may be an adaptive strategy for cooling, when outdoor temperatures exceed optimal temperatures. Prior studies of *Ae*. *aegypti* in desert climates have occurred in Texas, Arizona, and parts of Mexico [76,77]. In Mexico, a study found differences in the age structure of *Ae*. *aegypti* populations between two cities in desert and steppe climates, with older *Ae*. *aegypti* populations in the desert [76]. While precipitation did not differ much between the two sites, the cooler steppe population underwent a period of low humidity, which may impact survival. This is important for disease transmission, as *Ae*. *aegypti* must live long enough to feed and become infectious, and the authors suggested that the higher population turnover in the steppe may contribute to the surprising lack of dengue establishment. This points to the complexities of interactions between temperature (which can exceed optima for survival), precipitation, and sufficient humidity in an arid environment. Here, we were limited by a lack of available local humidity data;. given the arid conditions in Huaquillas, investigating the role that humidity plays in modulating mosquito presence and survival is a potential target for future studies.

In this study, the presence of *Ae*. *aegypti* at the household level differed across months. Changes in *Ae*. *aegypti* presence at coarse temporal scales are driven by climatic factors [78,79], and increased household *Ae*. *aegypti* presence in Huaquillas during the study period can be attributed to differences in precipitation and temperature. The seasonal nature of Huaquillas’ climate may thus point to time periods when targeted vector control interventions would be optimally effective.

### Social-ecological drivers of risk

In univariate models, we found that only factors related to infrastructure were significant predictors of *Ae*. *aegypti* presence. Interrupted water supply was the only statistically significant risk factor identified in this study, where having an unreliable water supply in the household was associated with increased risk of mosquito presence. The role of water infrastructure in exposure risk to *Ae*. *aegypti* at the household level has been found in previous studies, and points to the fundamental and vital role reliable water supply and urban infrastructure play in *Ae*. *aegypti* endemic environments [80]. Notably, the link between interruptions in water service and either *Ae*. *aegypti* presence or dengue fever risk has been documented previously in Ecuador, in the coastal city of Machala [38,80]. Unreliable water sources underpin many of the water storage behaviors that create suitable ovipositional sites for *Ae*. *aegypti*, such as when water for household activities (e.g. bathing, washing clothes, etc) is stored in open containers, such as buckets, drums, or basins. Further, the arid climate of Huaquillas may exacerbate water storing practices in absence of reliable piped water. Other studies in Latin American and the Caribbean have demonstrated links between drought conditions and increased mosquito abundance and dengue fever risk [22,81]. In these instances, extreme climatic events like drought drive water scarcity, which in turn promotes water storing practices, increasing the number of water containers in and around homes. While our study was conducted in an exceptionally dry year, the long-term arid climate of Huaquillas may promote greater water storage in absence of reliable water, compared to other locations in Ecuador. Given the clear role of water availability, our findings suggest that urban infrastructure around water supply and use is playing a large role in the risk of *Ae*. *aegypti* presence in the household in Huaquillas. These findings have implications for Ministry of Health mosquito control efforts, where emphasizing removal of standing water and features that collect water around homes may supplement existing outreach messaging. More broadly, long-term interventions that target and improve infrastructure, namely water reliability or water storage systems, may serve to mitigate these risk factors in the future [18].

While our threshold for statistical significance was set at α = 0.05, it is worth noting that the association between another infrastructural variable, biweekly trash collection (OR = 3.57, CI: 0.97–14.13), and mosquito presence approached statistical significance (p = 0.061). The association of mosquito risk with regular trash collection seems counterintuitive, where potential ovipositional sites for *Ae*. *aegypti* are reduced in the immediate environment. Yet, associations between garbage collection and *Ae*. *aegypti* presence or dengue fever were also observed in Machala and Guayaquil, two other coastal cities in Ecuador, possibly indicating that access to municipal services like trash collection may be a proxy indicator of built environments in Ecuador that are easily exploited by *Ae*. *aegypti* for reproduction [44,80].

In the single variable model, septic tanks were found to be protective against mosquito presence though marginally significant (p-value = 0.051, OR = 0.13 CI: 0.01–0.98). This is perhaps another counterintuitive finding, as septic tanks act as persistent oviposition sites in other locations [82–84]. Yet, it must be noted that all three households with septic tanks in this study were located in a single cluster. Additionally, septic tanks in Huaquillas are typically underground with no suitable entrance for mosquitoes, and are used widely in the periphery of the city where sewer infrastructure has only recently become available. Given that septic tanks occur in peripheral areas where municipal water and sewer infrastructure is a recent addition, this relationship warrants further examination.

## Conclusion

In this study we explored climatic and social-ecological factors associated with household-level female *Ae*. *aegypti* presence, and temporal and spatial trends across an arid border city in Ecuador. The results of our analyses may inform potential control strategies (timing) and interventions (improved water infrastructure) to reduce vector-borne disease risk in the city of Huaquillas in southern coastal Ecuador. Given that this study was conducted in an exceptionally dry year and the evidence for water supply and usage as major factors in household-level risk, water-related interventions at multiple scales could be important.

The social-ecological environment that influences the urban *Ae*. *aegypti* mosquito varies substantially from place to place. Local studies are especially needed to guide policy and inform interventions. Integrated vector control requires information collection, assessment, and decision making at local scales. While there is value in national to global studies of *Ae*. *aegypti* populations, these studies produce information that is of most relevance for national or international decision-makers. We acknowledge that there are logistical and resource related challenges inherent in conducting investigations at smaller scales, and in this study, while leveraging an immensely rich dataset over several months, we still ran into issues of small sample size. However, in order to integrate social-ecological systems approaches into essential local-scale work, we suggest that the design and methodological approach of this study is one example of how some of these challenges can be met. Identifying local risk factors for vector presence is critical for the successful control of mosquito-borne diseases, particularly in cities near international borders that serve as economic and transportation hubs, like Huaquillas, which has been the focus of binational efforts with Peru to control vector-borne diseases. As many parts of the world become increasingly urban and ever more connected to global transportation networks, the number of places with endemic *Ae*. *aegypti* populations will increase. Climate change throughout the 21st century is also set to increase the area suitable for *Ae*. *aegypti* presence [85]. These developments will further increase the importance of research at multiple scales to guide management and policy. Vector control will continue to be a critical component of arboviral disease prevention, even as additional intervention options become available. Understanding the systems which allow vectors to exist, persist, and transmit disease will remain critical in promoting human health and wellbeing for the foreseeable future.

## Supporting information

S1 Table The number of households with a given SES factor, shown by sampling clusters. Factors in bold were statistically significant in univariate analyses.
(DOCX)

S2 Table Matrix of *p* values from post hoc tests on pairs of months. (DOCX)

S1 Fig Spearman’s rank correlation values (rho) for climate variables and household *Ae*. *aegypti* presence, lagged up to five weeks. Asterisks denote significant lags.
(TIF)
