# Supplementary material for: Household and climate factors influence Aedes aegypti presence in the arid city of Huaquillas, Ecuador
Source: PLoS Negl Trop Dis. 2021 Nov 16;15(11):e0009931. doi: 10.1371/journal.pntd.0009931 (PMC8651121; doi:10.1371/journal.pntd.0009931)
Supplement: S1 Table — Factors in bold were statistically significant in univariate analyses. (DOCX) [file pntd.0009931.s001.docx]

**S1 Table. The number of households with a given SES factor, shown by sampling clusters. Factors in bold were statistically significant in univariate analyses.**

|  | **Sampling Clusters** | | | | | | | | | |
| --- | --- | --- | --- | --- | --- | --- | --- | --- | --- | --- |
| **SES Factor** | **1** | **2** | **3** | **4** | **5** | **6** | **7** | **8** | **9** | **10** |
| Good Overall Condition | 1 | 1 | 0 | 1 | 4 | 1 | 2 | 3 | 1 | 2 |
| Shaded Patio | 3 | 1 | 3 | 0 | 5 | 3 | 3 | 5 | 1 | 3 |
| Good Flooring | 2 | 1 | 0 | 1 | 1 | 2 | 0 | 3 | 1 | 3 |
| Screens on Windows | 0 | 0 | 0 | 0 | 2 | 1 | 1 | 2 | 0 | 1 |
| Abandoned Houses Nearby | 0 | 1 | 1 | 3 | 6 | 1 | 3 | 3 | 1 | 3 |
| Unpaved Road | 0 | 0 | 0 | 2 | 6 | 1 | 3 | 6 | 0 | 2 |
| **Water Interruptions** | 2 | 0 | 0 | 1 | 2 | 0 | 0 | 3 | 0 | 0 |
| **Uses Septic Tank** | 0 | 0 | 0 | 0 | 0 | 0 | 3 | 0 | 0 | 0 |
| Biweekly Trash Collection | 3 | 1 | 3 | 3 | 0 | 0 | 0 | 6 | 1 | 3 |
