# Supplementary material for: Household and climate factors influence Aedes aegypti presence in the arid city of Huaquillas, Ecuador
Source: PLoS Negl Trop Dis. 2021 Nov 16;15(11):e0009931. doi: 10.1371/journal.pntd.0009931 (PMC8651121; doi:10.1371/journal.pntd.0009931)
Supplement: S2 Table — (DOCX) [file pntd.0009931.s002.docx]

**S2 Table. Matrix of *p* values from post hoc tests on pairs of months.**

| Month | January | February | March | April | May |
| --- | --- | --- | --- | --- | --- |
| January |  |  |  |  |  |
| February | 0.254 |  |  |  |  |
| March | 0.449 | 0.824 |  |  |  |
| April | 0.725 | 0.449 | 0.725 |  |  |
| May | 0.725 | 0.139 | 0.215 | 0.449 |  |

Fisher’s exact test was conducted using the proportion of sampled households where *Aedes aegypti* was present. P values adjusted using the false discovery rate method [1].

**References**

1. Benjamini Y, Hochberg Y. Controlling the false discovery rate: a practical and powerful approach to multiple testing. Journal of the Royal statistical society: series B (Methodological). 1995;57: 289–300.
