# Supplementary figures and images for: Household and climate factors influence Aedes aegypti presence in the arid city of Huaquillas, Ecuador
Source: PLoS Negl Trop Dis. 2021 Nov 16;15(11):e0009931. doi: 10.1371/journal.pntd.0009931 (PMC8651121; doi:10.1371/journal.pntd.0009931)

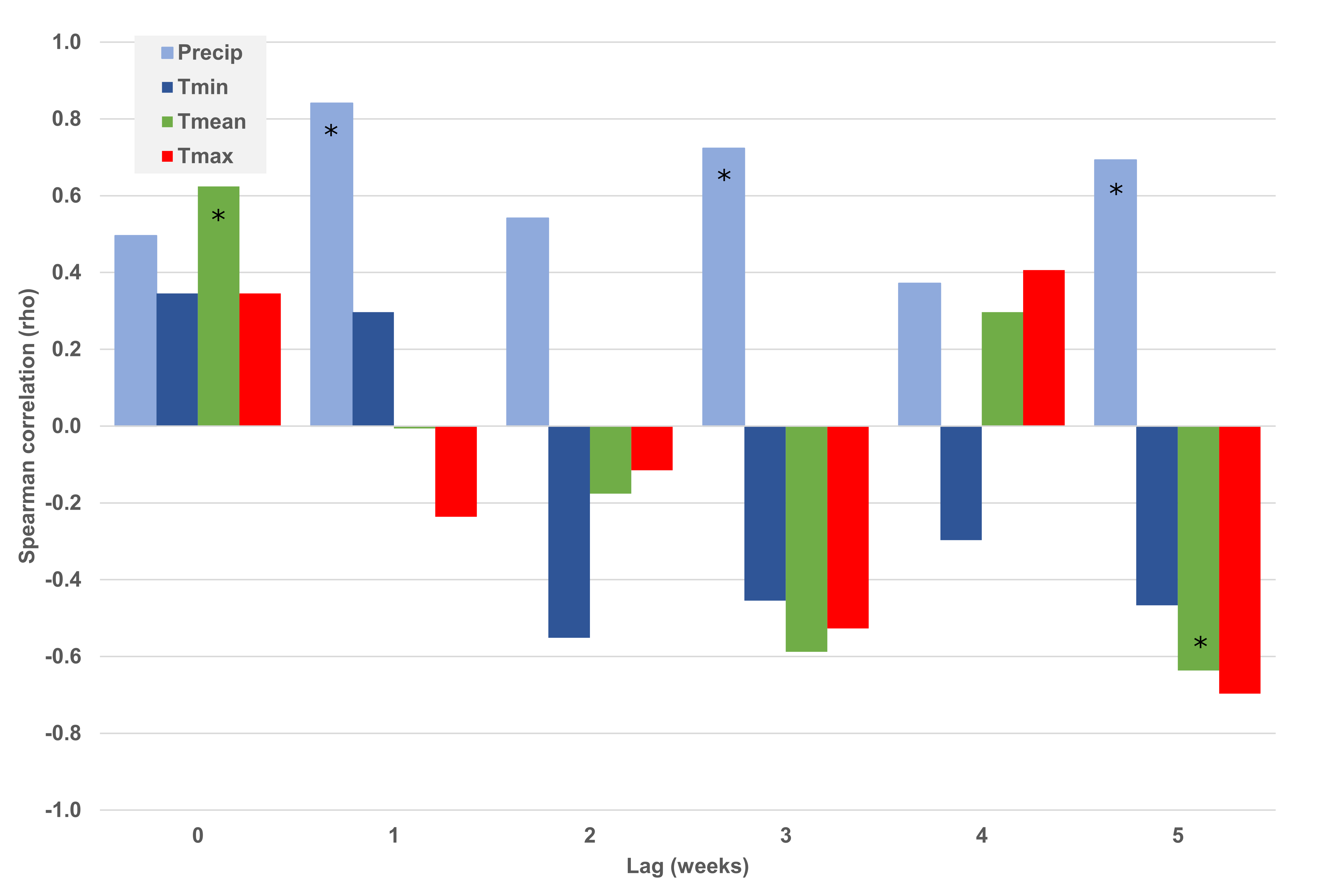

Supplement: S1 Fig — Asterisks denote significant lags. (TIF) [file pntd.0009931.s003.tif]
